# First review on the selenium status in Germany covering the last 50 years and on the selenium content of selected food items

**DOI:** 10.1007/s00394-022-02990-0

**Published:** 2022-09-09

**Authors:** Marina Liaskos, Nicole Fark, Paola Ferrario, Ann Katrin Engelbert, Benedikt Merz, Bernd Hartmann, Bernhard Watzl

**Affiliations:** 1 Department of Physiology and Biochemistry of Nutrition, Max Rubner-Institut (MRI) - Federal Research Institute of Nutrition and Food, Haid-und-Neu-Strasse 9, 76131 Karlsruhe, Germany; 2 Department of Nutritional Behaviour, Max Rubner-Institut (MRI) - Federal Research Institute of Nutrition and Food, Haid-und-Neu-Strasse 9, 76131 Karlsruhe, Germany

**Keywords:** Selenium status, German population, Selenium content of food, German nutrient database

## Abstract

**Introduction:**

Selenium is important for human health. However, the selenium status and selenium intake of the German population has not been recorded in a representative study so far.

**Material and Methods:**

Thus, literature from the last 50 years was screened in a systematic way and the results of various studies were pulled together to shed light on the selenium status of the German population. Moreover, the selenium content of selected food items that were either found on the German market or grown in Germany was researched and evaluated.

**Results:**

Of 3542 articles identified, 37 studies met the inclusion criteria. These 37 studies comprised a total of 8,010 healthy adults living in Germany with a weighted arithmetic mean of 82 μg/l selenium in plasma or serum. The results will form a basis for interpreting upcoming results from national food consumption surveys. Furthermore, 363 selenium values for 199 food items were identified out of 20 data sources—published or analysed between 2002 and 2019. An estimation of the selenium intake of the German population will be possible with this data in future nutrition surveys.

**Supplementary Information:**

The online version contains supplementary material available at 10.1007/s00394-022-02990-0.

## Introduction

The trace element selenium is important for the human health: both deficiency and excess will cause substantial health problems. Only 60 years ago selenium was recognised as being essential in the diet of animals and humans. As part of selenoproteins, selenium plays a role in anti-oxidant and anti-inflammatory regulations and in the production of active thyroid hormones [1]. In 2008 Bleys et al. described a U-shaped association of selenium status and mortality with the lowest mortality rate at a serum selenium concentration of 130–150 µg/l in American citizens [2]. The conditions that have been associated with selenium deficiency—commonly defined as plasma/ serum selenium concentrations < 20–40 µg/l—as well as selenium excess—commonly defined as plasma/ serum selenium concentrations > 140 µg/l—are listed in a recent review by Rayman [3].

So far there is no large, representative study on the selenium status of the German population, which makes it difficult to evaluate whether the people in Germany are adequately supplied. Only various smaller studies were carried out over the last decades. In the latest German National Nutrition Survey (NVS) II [4] the selenium intake of the population was not assessed due to missing data for selenium content of food items in the German Nutrient Database ‘Bundeslebensmittelschlüssel’ (BLS) (version 3.02). This database provides nutrient contents of around 15,000 food items found on the German market and serves as a standard instrument to calculate the nutrient intake in consumption surveys in Germany. Before including a new nutrient variable in the BLS, the data availability of this nutrient has to be proved to be sufficient for providing quality-checked data for all foods of the BLS. Due to the systematics of this nutrient database, nutrients cannot be included for a selection of foods only, because no missing values are allowed. Currently the BLS working group reviews whether in a future version selenium can be included or whether there will be a supplementary table for selenium for selected foods.

Unlike other minerals and trace elements selenium exists as part of various compounds with different bioavailabilities, metabolic pathways and half-lives. In food, selenium is mostly present as selenite, selenomethionine (SeMet), methylselenocysteine (MeSeCys) and selenocysteine (SeCyS)—or proteins containing these [5–7]. The selenium concentration of human and animal food varies widely between geographical areas as the uptake of selenium in plants depends on various factors [8–10]. In animal products the selenium content is influenced by the natural selenium content of the animal fodder and the amount of selenium added as feed additives [11].

In consequence, the selenium content in food is highly variable due to a number of particularities, making it challenging to state precise values of selenium content in specific food items. The literature search of selenium content of selected foods that were either found on the German market or grown in Germany discussed below was part of the BLS review process. With this approach the planned NVS III shall be enabled to provide representative information on the selenium status as well as the selenium intake of the German population.

Furthermore, literature from the last 50 years was reviewed in a systematic way to shed light on the selenium status of the German population and to form a basis to interpret new results from upcoming national nutrition surveys.

## Materials and methods

### Literature review on the selenium status of people living in Germany

#### Search strategy

The search strategy aimed to find published data on the selenium status of the German population from 1970 up until March 2021. It included articles obtained through Web of Science, Embase, Pubmed, Scopus, Cochrane, opengrey and grey literature searches. The search term used was (“Nutritional Status[Mesh]” OR “concentr*” OR “level*” OR “status”) AND (“Selenium[Mesh]” OR “selen*”) AND “german*” AND “human*”. The search term was adjusted to the different requirements of the used databases.

#### Data extraction

To provide reliable estimates for the selenium status in recent decades, number of study participants, mean values of selenium in serum/ plasma and corresponding standard deviations or standard errors, respectively, were extracted from all eligible studies. For studies that did not provide these numbers in the publication but seemed eligible—for instance selenium data were only included as a graph, the data was only stated as median or range, or a German subgroup needed to be extracted out of a pool of participants in a European data set—the corresponding authors were contacted. Data of healthy control groups were used when studies examined the selenium status in specific populations with a certain disease. Where possible the data for men and women were extracted separately.

#### Inclusion and exclusion criteria

Studies were included when they were published in English or German language as primary study to report the selenium status plus standard deviation/ standard error in blood plasma or blood serum of healthy individuals living in Germany. Information on the selenium status in whole blood samples was excluded for reasons of comparability. To obtain results of general relevance, only values from healthy adults over 18 years of age were used for the present review. Studies were excluded if they did not provide extractable data on selenium status in plasma or serum, only consisted of a study group of less than 10 participants or of pregnant/ nursing women.

Intervention studies specifically examining selenium supplementation were excluded or only control data used, as these studies often use pharmaceutical doses. Most observational studies did not provide specific information on selenium supplement use. However, some studies reported a general use of supplements. For example, Burney [12]: 22% of participants were taking vitamin or mineral supplements. These studies were still included for following reasons: First, no information was given on the exact mineral or vitamin taken, second, the use of supplements is widespread in the German population–about 28% of the adults in Germany are taking dietary supplements [4]. Including these studies gives a comprehensive estimate of the status of the German population.

Studies that only provided a mean value without standard deviation or standard error–even after contacting the author—were excluded as the accuracy of their reported results could not been assessed properly. No available risk of bias tool met the specific requirements of our study, thus an own quality assessment was performed for all included studies followed by a sensitivity analysis. The risk of bias was estimated based on the following quality measures: use of reference material/standards, performance of repeat measurements, accuracy of analysis, recovery rate, precision of analysis, limit of quantification LOQ / limit of detection LOD, performance of interlaboratory comparison, accreditation of the laboratory and the representativeness of the study population. All given parameters were considered together to rate the risk of bias as low, moderate or serious. Studies were classed as serious risk if they provided none or only one of the chosen quality measures. An exemption here are studies that were carried out by accredited laboratories as this implies that certain standards are fulfilled. Furthermore, studies that provided two or three of the chosen quality measures were classed as moderate risk, while studies with more than four given quality measures were classed as low risk of bias. A sensitivity analysis was then performed without the studies classified as serious risk of bias.

#### Statistical parameters

The quantitative data for selenium concentrations in blood serum or plasma is provided in µg/l. Where necessary the atomic weight of 78.97 was used to convert the extracted data to the used selenium unit. Data shown as standard error of the mean (SEM) in single studies were altered to standard deviation (SD) with the formula SD = SEM * √*n*. Range describes the smallest to the largest value of a given data set.

The weighted arithmetic mean was calculated with the following formula to account for the number of study participants with ω_i_ representing the weight (number of data points) associated with the sample (the selenium concentration) *X*_i_:

$$\frac{\sum_{i=1}^{n}{w}_{i}{X}_{i}}{\sum_{i=1}^{n}{w}_{i}}$$

The time period of sample collection often differed from the year of publication in the found studies. When known, the year of the examination or the midpoint of the examination period was used for the present study. Otherwise the publication date was used or authors were asked for detailed information.

### Literature review on the selenium content of food items

#### Search strategy

Selenium contents of food items were collected through literature search with Web of Science and Scopus. Pubmed and Embase were not included as these databases tend to have a rather medical focus. The search term consisted of "selenium" combined with various food items or food groups. Food items were selected if consumed frequently or in large quantities in Germany based on the results of the NVS II and its longitudinal equivalent NEMONIT. In addition, selenium rich food items such as Brazil nuts were included as they substantially contribute to the daily intake of selenium even if they are only consumed occasionally. Relevant publications were identified through scanning titles and available abstracts. Studies in English and German language were considered. Besides, reports of the German Federal Office of Consumer Protection and Food Safety (BVL) and the German Federal Institute for Risk Assessment (BfR) were screened.

#### Inclusion and exclusion criteria

Studies were selected when the investigated food samples were bought on the German market or grown in Germany. In addition, the samples had to refer to the edible part of the food item and it had to be clear which part of the food item was analysed, e.g., if the food item was peeled before processing. The data had to be based on precisely described analyses.

The quality of the collected data was evaluated based on an adapted quality index developed by the European Food Information Resource. The following categories were assessed: Food description, component identification, sampling plan, number of analytical samples, sample handling and analytical method. Data which received a low quality index were not considered [13].

#### Statistical parameters

Minimum, maximum, range and weighted arithmetic mean for the selenium content of foods are presented in µg/ 100 g. The applied weighting factor reflects the number of primary samples which were used in the selenium analyses. To show the relative contribution of specific foods to the reference value for selenium of the German Nutrition Society (DGE, 60 µg/day for women and 70 µg/day for men) [14], the weighted arithmetic mean of some collected food items is shown in relation to the selenium intake per portion and its contribution to the overall recommended selenium intake based on the reference value. The portion sizes of nuts are based on the food based dietary guidelines of the DGE [15]. For the other food items the quantity list “Monica Mengenliste” was preferably applied which was originated through the WHO Monica Study [16]. The list was compiled specifically for the calculation related to portion and household sizes, respectively. When no data were found the portion sizes of the BLS (version 3.02) were used.

## Results

### Selenium status in Germany

**Fig. 1:**
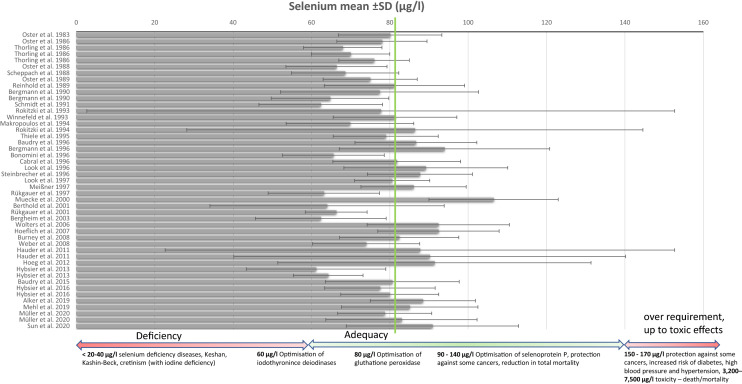
Mean of serum/ plasma concentration (µg/l) pulled from various studies reporting the selenium status of people in Germany over the last 50 years. Green line indicates weighted arithmetic mean of serum/ plasma selenium of all studies included (82 µg/l) for a total number of 8010 healthy adults living in Germany. Possible related health effects from deficiency to toxicity adapted from Fairweather-Tait et al. [5]

Of 3542 articles identified until March 2021, 37 studies with 47 data points were included in this review (see Fig. 1 and Table S1 for details). These 37 studies comprised a total of 8010 healthy adults living in Germany with a mean selenium concentration in plasma or serum of 79 μg/l, ranging from 61 μg/l to 107 μg/l. To account for the number of participants per study, the weighted arithmetic mean of 82 μg/l selenium in plasma or serum for the 8010 healthy adults living in Germany was calculated.

Based on the risk of bias assessment, 10 studies were evaluated to have a serious risk of bias, whereas all other studies showed either a moderate (*n* = 22) or low (*n* = 5) risk of bias (Table S2). In a sensitivity analysis where we excluded all studies with a serious risk of bias, the arithmetic mean was robust and remained at 82 μg/l selenium in plasma or serum. Therefore, all studies were included to have a wider range of data points distributed over the last decades.

No study provided representative results to the German population since none of the included studies applied a representative, population-based sampling procedure when recruiting study participants.

Mean serum/ plasma selenium concentration for men was available from 16 studies and the arithmetic mean was 80 μg/l (range: 60–90 μg/l) for 2256 men. Mean serum/ plasma selenium concentration for women was available from 14 studies and the arithmetic mean was 84 μg/l (range: 64–94 μg/l) for 4,124 women.

The literature was screened from 1970 to March 2021 with the earliest study found dating back from 1983. Investigations from 1983 to 1990 showed an arithmetic mean of serum/ plasma selenium concentration of 74 μg/l, from 1991 to 2000 of 83 μg/l, from 2001 to 2010 of 73 μg/l and from 2011 to 2020 of 88 μg/l.

### Selenium content of food items

Out of 20 data sources 363 selenium contents for 199 different food items were identified (see Table S3). The data were published or analysed between 2002 and 2019. Before 2002 no eligible publication could be found. The majority of selenium data was collected from the BVL National Monitoring (2002–2019). Most data were available for vegetables (84 values for 36 different food items), fruits (54 values for 30 food items), meat and meat products (45 values for 25 food items) and fish and seafood (33 values for 19 food items).

The Brazil nut is the food item with the highest selenium content with a weighted arithmetic mean of 277 µg/ 100 g. It is followed by offal: pork kidney 221 µg/ 100 g (range 220–223 µg/ 100 g), beef kidney 123 µg/ 100 g (range 118–154 µg/ 100 g) and veal kidney 98.6 µg/ 100 g. Furthermore, the food group “fishes and seafood” shows notable selenium contents. The highest content (71.7 µg/ 100 g, range 63.9–81.8 µg/ 100 g) in this group was reported for tuna.

The contribution of different food items to achieve the reference value of selenium intake varies widely (Table 1). As a calculation example the recommended daily intake of 70 µg for men and 60 µg for women can be covered with a portion (125 g) of pork liver (100% for men and 116% for women) or with a handful (25 g) of Brazil nuts (99% or 115%, respectively). 154% (men) and 179% (women) of the recommended intake will be achieved with a portion of tuna (150 g). The highest intake per portion (125 g) provides pork kidney (395% or 461%, respectively). Against these food items, an apple (115 g) can only obtain 3% (men) and 4% (women) to the recommended daily selenium intake. A contribution between 17 to 32% of these recommendations can be covered with a portion of eggs, mushrooms or meat (beef, pork, veal).

**Table 1:** Overview of collected selenium data of various food items, selenium intake per portion with the percentage of daily intake recommendation for men and women

Food group	Food item	Number of primary samples	Weighted arithmetic mean (µg/ 100 g)	Minimum (µg/ 100 g)	Maximum (µg/ 100 g)	Range (µg/ 100 g)	Portion size (g)	Intake (µg/portion)	Recommended intake (%/portion)		References
									Men	Women	
Cereals	Oat	247	8.69	7.24	9.80	2.56	60	5.21	7	9	[17, 18]
	Rice unpolished	151	4.71	4.62	4.82	0.20	60	2.83	4	5	[17, 19]
	Rye whole grain	214	2.73	1.85	4.25	2.40	40	1.09	2	2	[20–22]
	Wheat whole grain	393	7.70	4.35	37.1	32.8	40	3.08	4	5	[23–27]
Seeds	Linseed	201	19.8	12.6	30.3	17.7	25	4.95	7	8	[20, 28, 29]
	Sesame	188	39.0	34.9	43.6	8.76	25	9.75	14	16	[30, 31]
	Sunflower seed	128	17.6	10.5	18.9	8.44	25	4.40	6	7	[23, 32]
Nuts	Brazil nut	71	277				25	69.1	99	115	[23]
	Hazelnut	15	40.6				25	10.1	14	17	[32]
	Peanut roasted	241	36.8	14.7	47.4	32.7	25	9.19	13	15	[30–33]
Fruits	Apple	121	1.85	0.15	2.00	1.85	115	2.12	3	4	[18, 21]
	Banana	358	1.37	0.97	2.00	1.03	100	1.37	2	2	[25, 28, 34]
Vegetables	Carrot	483	2.25	0.22	6.92	6.70	80	1.80	3	3	[17, 29–32, 34]
	Garlic	51	2.63				2	0.05	0	0	[17]
	Lamb´s lettuce	171	1.22	0.42	1.35	0.93	50	0.61	1	1	[17, 30, 31, 33]
Mushrooms	Mushroom	196	11.7	9.08	13.0	3.92	100	11.7	17	19	[22, 24]
Potato	Potato peeled	238	1.00	0.90	1.10	0.20	200	2.01	3	3	[17, 32]
Fat and oil	Olive oil	96	2.29				10	0.23	0	0	[24]
Milk and dairy products	Cream cheese	57	2.76	< LOQ	2.81	2.81	15	0.41	1	1	[18]
	Gouda	176	15.4	14.6	16.1	1.44	30	4.60	7	8	[17, 18]
	UHT-Milk (3.8%)	122	1.44				150	2.16	3	4	[18]
Eggs	Egg	153	22.1	21.1	24.2	3.12	55	12.2	17	20	[24, 26]
Meat and meat products	Beef	629	8.00	6.51	8.69	2.18	150	12.0	17	20	[17, 22, 28, 34]
	Pork	207	12.6	11.3	13.6	2.39	150	19.0	27	32	[19, 21]
	Veal	304	9.50	7.66	10.3	2.63	150	14.3	20	24	[18, 24]
	Lyoner sausage	97	7.28				20	1.46	2	2	[32]
	Salami	151	17.5				20	3.50	5	6	[29]
Offal	Beef liver	284	35.2	33.6	38.3	4.76	125	44.0	63	73	[17, 26]
	Pork liver	164	55.9	51.3	64.1	12.8	125	69.8	100	116	[19, 20, 26]
	Pork kidney	156	221	220	223	3.54	125	276	395	461	[18, 26]
Fish	Crab	134	45.4	41.3	48.4	7.13	100	45.4	65	76	[18, 24, 32]
	Salmon	227	15.7	13.5	18.6	5.1	150	23.6	34	39	[17, 29]
	Rainbow trout	220	15.6	12.2	18.8	6.67	150	23.4	33	39	[23, 32]
	Tuna	226	71.7	63.9	81.8	17.9	150	107	154	179	[26, 28, 31]
	Trout smoked	63	19.7				75	14.8	21	25	[32]
Beverages	Beer	318	1.34	1.17	2.00	0.83	330	4.44	6	7	[22, 34]
	Orange juice	313	0.83	0.49	1.30	0.81	200	1.66	2	3	[25, 28, 33]
Sweets	Chocolate	13	32.6				20	6.53	9	11	[32]
	Dark chocolate	259	10.9	8.06	13.8	5.76	20	2.18	3	4	[24, 26]

*Blanks mean no data available

## Discussion

### Selenium status of the German population

This is the first time that data from various studies were combined in a systematic way to estimate the selenium status of the German population. Pulling together the available data indicates an average selenium concentration of 82 μg/l (weighted arithmetic mean) in plasma or serum for a total number of 8010 healthy adults living in Germany. Even though no study representative to the whole German population could be included in the present review, the combination of many different subgroups gives an idea of the approximate selenium status of the German population.

A comprehensive review by Fairweather-Tait et al. [5] concluded that an adequate selenium status lies within the range of 60 to 140 μg/l in serum or plasma. The authors combined various associations of selenium status and health parameters from the published literature. Deficiency diseases are shown with a selenium level under 20–40 μg/l – for example, Keshan disease or Kashin–Beck disease. A selenium status over 65 μg/l leads to an optimisation of iodothyronine deiodinases activity, and over 79–124 μg/l to an optimisation of glutathione peroxidase and selenoprotein P activity [35–37]. Further studies suggest a reduction in total mortality or protection against some cancers [5, 38, 39]. In contrast, a Cochrane review including 10 randomized controlled trials (with a total of 27,232 participants) and 70 observational studies (with over 2,360,000 participants) from 2018 investigated the effect of selenium exposure on cancer risk and did not observe a beneficial effect of selenium supplements on cancer risk [40]. Toxic effects are seen from around 150 μg/l [5, 35]. As mentioned in the introduction, Bleys et al. [2] presented the U-shaped relationship between selenium and mortality—the optimal selenium status with the lowest mortality risk was shown between 130 and 150 μg/l. 13,887 adult participants were examined and followed up for 12 years as part of the US Third National Health and Nutrition Examination Survey for this study [2]. These results were verified by Goyal et al. [41]. However, they cannot be generalised to the rest of the world as they were obtained in North America, where the average daily intake of selenium is considerably higher compared to other countries due to the higher selenium content of foods as a result of various reasons such as selenium content of the soil, pH of the soil and other factors [8].

The U-shaped relationship between selenium status and its health effects might not be sufficient to explain the interindividual differences in the effect of selenium on health. Different requirements might exist in populations and individuals. Rayman [3] recently discussed four reasons for the fact that certain populations and people have different selenium requirements and can tolerate low or high selenium intakes better than others:

1. Single nucleotide polymorphisms in genes might improve the ability to metabolise high or low selenium.
2. Populations exposed to harmful elements show no or even beneficial effects of high selenium status. The Inuit in Canada are, for example, exposed to high amounts of mercury as their diets consist primarily of marine mammals and fish. They also show a high mean blood selenium level of 319.5 μg/l. The high selenium status proved to have protective effects on odds ratios for hypertension, stroke and myocardial infarction associated with mercury exposure.
3. The dose and form of selenium determines if the overall effect is beneficial or harmful.
4. The gut microbiota might have a regulating function. Exposure to high amounts of selenium in early life might, for example, alter the composition of the gut microbiota in a way that selenium is more readily excreted.

#### Variation of the selenium status over the last 40 years

The earliest studies found in this literature search were conducted in the 1980s. The selenium status over the last 40 years does not follow an apparent pattern. The variations might be due to changes of available food items on the market. An example would be the effect of a reduced import of North American wheat for breadmaking flour in the 1970s, which might have negatively influenced the selenium status in some populations in the European regions [42] or the increase of serum selenium concentrations in women living in Dresden after the German reunification seen by Bergmann et al. [43]. Seasonal differences of the selenium status might also influence the results as seasonal fluctuations of selenium have been shown in plants accumulating selenium [44]. Many other factors influence how selenium moves through the soil–plant–atmosphere interface [45] and might impact the selenium status of a population.

Another reason could be the different age ranges used in the studies. A decline of selenium bioavailability was shown to occur with increasing age by Olivieri [46] and Baudry [47]. Furthermore, differences in the selenium supply could occur due to environmental influences on the selenium uptake by plants. Rayman [42] discussed a possible influence of acid rain or excessive artificial fertilisation of the soil, which reduces plant absorption of selenium. Jones et al. [48] highlighted the importance of the soil–plant interactions as main factor controlling selenium bioavailability in the human diet and predicted a substantial decrease of soil selenium particularly in agriculture areas until 2080–2099 using moderate climate change predictions.

#### Gender differences

Arithmetic mean of the selenium status was 80 μg/l for 2,256 men and 84 μg/l for 4124 women. The results are comparable to the results of the EPIC cohort [38] and both values are within the range of adequate supply according to Fairweather-Tait et al. [5].

However, a few studies suggest that women are more susceptible to the onset of certain health conditions associated with low or high selenium levels. A study by Arnaud et al. [49] implies that in women, the onset of metabolic syndrome is associated with higher plasma selenium concentrations but not in men. Hughes et al. [38] demonstrated a significant association between lower selenium levels and the risk to develop colorectal cancer in women, while this association was not significant for men.

### Variation of selenium contents in food items

Considering all the facts discussed so far, it becomes clear that it is an enormous challenge to integrate selenium data in nutrient databases, such as the BLS. The selenium content in plant-based foods varies due to considerable differences in the soil selenium. Meat products are affected through the selenium content of the plants/ soils plus the selenium supplementation of specific fodder [11]. Therefore, data of other parts of the world are not applicable for Germany which makes it difficult to compose representative data.

The high variations in the selenium content in one and the same plant species can be clearly demonstrated using the Brazil nut which is characterised by a naturally high selenium content [50]. The selenium content of the Brazil nut is by far the highest in this literature research with a weighted arithmetic mean of 277 µg/ 100 g. This result is similar to the analyses of Moreda-Piñeiro [51] with 254 µg/ 100 g in samples originating from Spain and Barclay et al. [52] with 300 µg/ 100 g in samples originating from the UK. However, in comparison to other studies these values are low: Three Brazilian studies report contents from 2270 to 3370 µg/ 100 g [53–55]. On the other hand, a value of 78.5 µg/ 100 g for Brazil nuts from Vietnam was found [56].

Thomson et al. [50] emphasized the importance of these nuts as a relevant selenium source. The authors clarified that the daily consumption of two Brazil nuts (average weight of 4.1 g per nut with a defined selenium content of 640 µg/ 100 g) has a notable impact of the selenium status. Due to the high variance of the amount of selenium in these nuts it is, however, difficult to give an exact recommendation and an over-consuming of selenium could be possible especially with the consumption of those Brazil nuts with higher selenium contents. The DGE recommends a handful of mixed nuts per day which corresponds to a portion of 25 g [15].

Meat is also a substantial selenium source. Through the supplementation of fodder, meat has considerable selenium contents even in countries with a selenium poor ground [11]. In Austria Stückler et al. [11] analysed meat with similar results to this review: beef 8.5 µg/ 100 g (8.00 µg/ 100 g) and pork 15.4 µg/ 100 g (12.6 µg/ 100 g). Offal contain high levels of selenium, but are rarely consumed nowadays. Furthermore, the food items of the group fish and seafoods such as mussels or crabs have comparably high selenium contents [18, 20, 24, 32]. A regular fish consumption may, therefore, contribute to an adequate selenium status. The DGE [15] recommends to consume fish one or two times a week. In contrast, vegetables and fruits are rather negligible selenium sources. In general, animal-based food items show higher selenium contents than plant-based food items.

The implementation of these literature-based data in the nutrient database will offer the opportunity to select the type and calculate the amount of food items necessary to achieve the reference values for selenium intake in Germany. Furthermore, this will support future consumption studies—such as the NVS III—to estimate the actual selenium intake of the German population and to evaluate which foods significantly contribute to the selenium intake in Germany.

## Conclusions

German nutrition surveys have not covered the selenium intake of participants in the past, since the data on selenium content of food items in applicable nutrient databases were not available for methodical reasons. Therefore, literature from 1970 up until March 2021 was reviewed in a systematic way to display the selenium status of the German population over this time span and to form a basis to interpret new results. Considering our results and the present literature, the majority of adults in Germany seem to be adequately supplied with selenium. However, even if the weighted arithmetic mean of the 37 investigated studies indicates an acceptable selenium status of 82 µg/l in plasma/ serum for a total of 8,010 healthy adults living in Germany, most likely not everyone in Germany will be sufficiently supplied. The planned NVS III with over 10,000 participants will provide highly needed information on the selenium status of the German population in general and of various subgroups (e. g. those following a vegan or vegetarian diet) by determining selenium in blood samples.

Furthermore, the selenium content of selected food items that were either found on the German market or grown in Germany was researched and evaluated. The availability of selenium data offers the opportunity to include those in the German Nutrient Database and to estimate the selenium intake of the German population as well as to identify the major selenium sources in future nutrition surveys such as the mentioned NVS III. Moreover, we will be able to further research the link between selenium intake and selenium status in the planned study.

## Supplementary Information

Below is the link to the electronic supplementary material.

Supplementary file1 (DOCX 49 KB)

## Data Availability

Not applicable.
