# Supplementary material for: First review on the selenium status in Germany covering the last 50 years and on the selenium content of selected food items
Source: Eur J Nutr. 2022 Sep 9;62(1):71–82. doi: 10.1007/s00394-022-02990-0 (PMC9899741; doi:10.1007/s00394-022-02990-0)
Supplement: Supplementary file 1 — Supplementary file1 (DOCX 49 KB) [file 394_2022_2990_MOESM1_ESM.docx]

Table S1: Overview of the included studies used to determine the selenium status of the German population by year of investigation/ publication

| year of investigation/ publication | authors | location | number of participants/ controls | gender | average age (mean ±SD or range) | BMI (mean ±SD) | measuring technique | sample | mean selenium conc. (µg/l) | ±SD | CI |
| --- | --- | --- | --- | --- | --- | --- | --- | --- | --- | --- | --- |
| 1983 | Oster et al. [57] | Mainz | 92 | 48 women, 44 men | 36.3 (16-61) |  | AAS | serum | **80** | 13 | 83 |
| 1986 | Oster et al. [58] | Mainz | 41 | men | 52 ±6 |  | AAS | serum | **78** | 12 | 82 |
| 1986 | Thorling et al. [59] | Giessen | 19 | equal number | (20 - 65) |  | TXRF | serum | **68** | 10 | 72 |
| 1986 | Thorling et al. [59] | Bavaria | 40 | equal number |  |  | TXRF | serum | **70** | 10 | 73 |
| 1986 | Thorling et al. [59] | Heidelberg | 23 | equal number |  |  | TXRF | serum | **76** | 9 | 80 |
| 1988 | Oster et al. [60] | Mainz | 72 | 47 women, 25 men | 36.3 ±13.3 |  | AAS | serum | **66** | 13 | 69 |
| 1988 | Scheppach et al. [61] | Würzburg | 39 | 17 women, 22 men | 21-71 |  | AAS | serum | **69** | 14 | 73 |
| 1989 | Oster et al. [62] | Mainz | 143 | 36 women, 107 men | 53 ±8 |  | AAS | serum | **75** | 12 | 77 |
| 1989 | Reinhold et al. [63] | Bonn | 80 | 38 women, 42 men | 51 (19 - 83) |  | AAS | serum | **81** | 18 | 85 |
| 1990 | Bergmann et al. [43] | Dresden | 229 | women | 50.59 ±5.31 | 24.41 ±3.62 | AAS | serum | **77** | 25 | 81 |
| 1990 | Bergmann et al. [43] | Dresden | 116 | men | 43.68 ±11.28 | 25.08 ±3.08 | AAS | serum | **65** | 15 | 67 |
| 1991 | Schmidt et al. [64] | Frankfurt | 20 | 9 women, 11 men | 18-55 |  | AAS | plasma | **62** | 16 | 69 |
| 1993 | Rokitzki et al. [65] | Freiburg | 13 |  | 34,6 ±9,8 |  | AAS | plasma | **78** | 75 | 118 |
| 1993 | Winnefeld et al. [66] | Jena | 100 |  |  |  | AAS | serum | **81** | 16 | 84 |
| 1994 | Makropoulos et al. [67] | Aachen | 261 | women |  |  | AAS | serum | **70** | 16 | 72 |
| 1994 | Rokitzki et al. [68] | Freiburg | 12 | men | 41.6 ±9.8 |  | AAS | plasma | **86** | 58 | 119 |
| 1995 | Thiele et al. [69] | Jena | 62 |  |  |  | AAS | serum | **79** | 13 | 82 |
| 1996 | Baudry et al. [47] | Potsdam | 219 | 88 women, 131 men | 58.32 ±4.42 | 26.72 ±3.50 | ICP-MS | serum | **87** | 16 | 89 |
| 1996 | Bergmann et al. [43] | Dresden | 361 | women | 50.59 ±5.51 | 25.14 ±4.15 | AAS | serum | **94** | 27 | 97 |
| 1996 | Bonomini et al. [70] | Rostock | 88 | 41 women, 47 men | 37.2 ±11.9 |  | AAS | plasma | **66** | 13 | 68 |
| 1996 | Cabral et al. [71] | Potsdam | 2248 | 1379 women, 869 men |  |  | ICP-MS | serum | **82** | 16 | 82 |
| 1996 | Look et al. [72] | Bonn | 72 |  |  |  | AAS | serum | **89** | 21 | 94 |
| 1996 | Steinbrecher et al. [73] | Heidelberg | 490 | men | 58.1 ±4.8 | 27.3 ±3.4 | ICP-MS | serum | **88** | 13 | 89 |
| 1997 | Look et al. [74] | Bonn | 48 | 16 women, 32 men | 40.2 (25-55) |  | AAS | serum | **81** | 10 | 83 |
| 1997 | Meißner [75] | Dresden | 256 | 198 women, 58 men |  |  | AAS | serum | **86** | 13 | 88 |
| 1997 | Rükgauer et al. [76] | Stuttgart | 68 |  | (22 - 75) |  | AAS | plasma | **63** | 14 | 67 |
| 2000 | Muecke et al. [77] | Münster | 75 |  |  |  | TXRF | serum | **107** | 17 | 110 |
| 2001 | Berthold et al. [78] | Cologne | 412 | 228 women, 184 men | 50.2 ±13.6 | 25.5 ±4.0 | ICP-MS | serum | **64** | 30 | 67 |
| 2001 | Rükgauer et al. [79] | Stuttgart | 99 | 54 women, 45 men | 37.4 ±11.7 | 23.9 ±3.6 | AAS | plasma | **66** | 8 | 68 |
| 2003 | Bergheim et al. [80] | Stuttgart | 22 | men | 45 ±11 | 24.4 ±2 | AAS | plasma | **62** | 17 | 69 |
| 2006 | Wolters et al. [81] | Hannover | 167 | women |  |  | AAS | serum | **92** | 18 | 95 |
| 2007 | Hoeflich et al. [82] | Berlin | 53 | equal number | 25.3 ±5.4 | 18 - 24.9 | TXRF | serum | **92** | 15 | 97 |
| 2008 | Burney et al. [12] | Berlin | 33 | 9 women, 24 men | 39 |  | AAS | plasma | **82** | 15 | 88 |
| 2008 | Weber et al. [83] | Bonn | 11 | 5 women, 6 men | median: 34 |  | AAS | serum | **74** | 14 | 82 |
| 2011 | Hauder et al. [84] | Karlsruhe | 25 | men | 62.6 ±7.4 | 27.4 (4.2) | AAS | serum | **88** | 65 | 113 |
| 2011 | Hauder et al. [84] | Karlsruhe | 25 | men | 63.6 ±6.4 | 27.8 (3.5) | AAS | serum | **90** | 50 | 110 |
| 2012 | Hoeg et al. [85] | Kiel + Berlin | 1083 | women |  |  | TXRF | serum | **91** | 40 | 94 |
| 2013 | Hybsier et al. [86] | Berlin | 20 | men | n=10 (22-26) and n=10 (73-79) |  | TXRF | serum | **61** | 18 | 69 |
| 2013 | Hybsier et al. [86] | Berlin | 20 | women | n=10 (22-26) and n=10 (73-79) |  | TXRF | serum | **64** | 9 | 68 |
| 2015 | Baudry et al. [47] | Potsdam | 219 | 88 women, 131 men | 77.6 ±3.93 | 27.13 ±4.06 | ICP-MS | serum | **81** | 17 | 83 |
| 2016 | Hybsier et al. [86] | commercial supplier | 43 | men | (18-64) |  | TXRF | serum | **78** | 14 | 82 |
| 2016 | Hybsier et al. [86] | commercial supplier | 56 | women | (18-55) |  | TXRF | serum | **80** | 13 | 83 |
| 2019 | Alker et al. [87] | commercial supplier | 154 | 79 women, 75 men |  |  | ICP-MS | serum | **88** | 13 | 91 |
| 2019 | Mehl et al. [88] | commercial supplier | 200 | equal number |  |  | TXRF | serum | **85** | 17 | 88 |
| 2020 | Müller et al. [89] |  | 25 | 19 women, 6 men | 58 ±13 (33-76) |  | ICP-MS | serum | **79** | 12 | 83 |
| 2020 | Müller et al. [89] |  | 26 | 17 women, 9 men | 61 ±11 (32 - 76) |  | ICP-MS | serum | **83** | 19 | 90 |
| 2020 | Sun, Q. et al. [90] |  | 30 | 19 women, 11 men | 53 (23-60) |  | TXRF | serum | **90** | 22 | 98 |

* blanks mean no data available, TXRF = total reflection x-ray fluorescence

Table S2: Quality assessment of included studies

| authors | method | reference material / standards | repeat measurement | accuracy of analysis | mean recovery rate | precision of analysis (coefficient of variation (CV)) | limit of quantification LOQ (µg/l)/ limit of detection LOD (µg/l) | interlaboratory comparisons | accreditation of the laboratory | representative study population | overall risk of bias |
| --- | --- | --- | --- | --- | --- | --- | --- | --- | --- | --- | --- |
| Alker et al. [87] | ICP-MS | Y | triplicate | Y | N/A | Y | 0.13 / 0.04 | N/A | N/A | no | **low** |
| Baudry et al. [47] | ICP-MS | Y | N/A | N/A | N/A | N/A | 10-sigma / 3-sigma criterion | N/A | N/A | no | **moderate** |
| Bergheim et al. [80] | AAS | Y | duplicate | N/A | N/A | N/A | N/A | N/A | N/A | no | **moderate** |
| Bergmann et al. [43] | AAS | Y | N/A | Y | N/A | Y | N/A | Y | N/A | no | **low** |
| Berthold et al. [78] | ICP-MS | Y | triplicate | N/A | N/A | CV 3-5% | N/A | N/A | N/A | no | **moderate** |
| Bonomini et al. [70] | AAS | Y | duplicate | Y | N/A | CV 1% | N/A / 1.00 | N/A | N/A | no | **low** |
| Burney et al. [12] | AAS | Y | duplicate | 6% | N/A | inter- and intraassay CV <10% | N/A / 1.60 | N/A | Y | no | **low** |
| Cabral et al. [71] | ICP-MS | Y | N/A | N/A | 97.3 ± 7.9% | N/A | 10-sigma / 3-sigma criterion | N/A | N/A | no | **moderate** |
| Hauder et al. [84] | AAS | N/A | N/A | N/A | N/A | N/A | N/A | N/A | Y | no | **moderate** |
| Hoeflich et al. [82] | TXRF | Y | triplicate | N/A | N/A | interassay CV <10% | N/A | N/A | N/A | no | **moderate** |
| Hoeg et al. [85] | TXRF | Y | N/A | N/A | N/A | inter- and intraassay CV <10% | N/A | N/A | N/A | no | **moderate** |
| Hybsier et al. [86] | TXRF | N/A | N/A | N/A | N/A | N/A | N/A | N/A | N/A | no | **serious** |
| Look et al. [72] | AAS | N/A | N/A | N/A | N/A | N/A | N/A | N/A | N/A | no | **serious** |
| Look et al. [74] | AAS | N/A | N/A | N/A | N/A | N/A | N/A | N/A | N/A | no | **serious** |
| Makropoulos et al. [67] | AAS | Y | N/A | N/A | N/A | N/A | N/A / 5.00 | N/A | N/A | no | **moderate** |
| Mehl et al. [88] | TXRF | Y | triplicate | N/A | N/A | interassay CV <10% | N/A | N/A | N/A | no | **moderate** |
| Meißner [75] | AAS | Y | N/A | N/A | N/A | CV 3.3% | N/A | Y | N/A | no | **moderate** |
| Muecke et al. [77] | TXRF | N/A | N/A | N/A | N/A | N/A | N/A | N/A | N/A | no | **serious** |
| Müller et al. [89] | ICP-MS | Y | N/A | N/A | N/A | N/A | 10-sigma / 3-sigma criterion | N/A | N/A | no | **moderate** |
| Oster et al. [57] | AAS | Y | duplicate | N/A | N/A | N/A | N/A | N/A | N/A | no | **moderate** |
| Oster et al. [58] | AAS | Y | duplicate | N/A | N/A | N/A | N/A | N/A | N/A | no | **moderate** |
| Oster et al. [60] | AAS | Y | duplicate | N/A | N/A | N/A | N/A | N/A | N/A | no | **moderate** |
| Oster et al. [62] | AAS | Y | duplicate | N/A | N/A | N/A | N/A | N/A | N/A | no | **moderate** |
| Reinhold et al. [63] | AAS | Y | N/A | N/A | N/A | N/A | N/A | N/A | N/A | no | **serious** |
| Rokitzki et al. [65] | AAS | Y | N/A | N/A | N/A | CV 5.6 % | N/A | Y | N/A | no | **moderate** |
| Rokitzki et al. [68] | AAS | Y | N/A | N/A | N/A | CV 5.6 % | N/A | Y | N/A | no | **moderate** |
| Rükgauer et al. [76] | AAS | Y | N/A | ±5% | N/A | precision value 1.50 µmol/l | N/A / 0.06 µmol/l | Y | N/A | no | **low** |
| Rükgauer et al. [79] | AAS | Y | N/A | N/A | N/A | N/A | N/A | Y | N/A | no | **moderate** |
| Schmidt et al. [64] | AAS | N/A | duplicate | N/A | N/A | N/A | N/A | N/A | N/A | no | **serious** |
| Scheppach et al. [61] | AAS | N/A | N/A | N/A | N/A | N/A | N/A | N/A | N/A | no | **serious** |
| Steinbrecher et al. [73] | ICP-MS | Y | N/A | N/A | N/A | inter- and intraassay CV <10% | N/A / 0.02 µmol/l | N/A | N/A | no | **moderate** |
| Sun et al. [90] | TXRF | Y | N/A | N/A | N/A | inter- and intraassay CV <10% | N/A | N/A | N/A | no | **moderate** |
| Thiele et al. [69] | AAS | N/A | N/A | N/A | N/A | N/A | N/A | N/A | N/A | no | **serious** |
| Thorling et al. [59] | AAS | N/A | N/A | N/A | N/A | N/A | N/A | N/A | N/A | no | **serious** |
| Weber et al. [83] | AAS | N/A | N/A | N/A | N/A | N/A | N/A | N/A | N/A | no | **serious** |
| Winnefeld et al. [66] | AAS | Y | N/A | N/A | 98.9-103% | intraassay CV 3.8% | N/A | N/A | N/A | no | **moderate** |
| Wolters et al. [81] | AAS | Y | N/A | N/A | N/A | intraassay CV 6% | N/A / 0.04 µmol/l | N/A | N/A | no | **moderate** |

Table S3: Collected selenium contents of food items through literature research

| **Food group** | **Food item** | **Number of primary samples** | **Weighted arithmetic mean (µg/ 100 g)** | **Minimum (µg/ 100 g)** | **Maximum (µg/ 100 g)** | **Range (µg/ 100 g)** | **Reference** |
| --- | --- | --- | --- | --- | --- | --- | --- |
|  |  |  |  |  |  |  |  |
| Bakery product | Pastry | 65 | 1.92 |  |  |  | [29] |
| Cereal and cereal products | Barley whole grain | 80 | 3.41 |  |  |  | [20] |
|  | Buckwheat | 179 | 3.82 | 3.09 | 4.46 | 1.37 | [30,31] |
|  | Bulgur | 89 | 3.49 |  |  |  | [25] |
|  | Cornmeal | 84 | 4.18 |  |  |  | [24] |
|  | Oat | 247 | 8.69 | 7.24 | 9.80 | 2.56 | [17,18] |
|  | Rice polished | 220 | 5.59 | 4.42 | 7.25 | 2.83 | [17,19] |
|  | Rice unpolished | 151 | 4.71 | 4.62 | 4.82 | 0.20 | [17,19] |
|  | Rye whole grain | 214 | 2.73 | 1.85 | 4.25 | 2.40 | [20-22] |
|  | Spelt whole grain | 164 | 5.34 | 5.10 | 5.63 | 0.53 | [24,28] |
|  | Wheat bran | 92 | 7.75 |  |  |  | [17] |
|  | Wheat whole grain | 393 | 7.70 | 4.35 | 37.1 | 32.8 | [23-27] |
| Seeds | Linseed | 201 | 19.8 | 12.6 | 30.3 | 17.7 | [20,28,29] |
|  | Pine nut | 206 | 3.51 | 3.21 | 3.82 | 0.61 | [24,30] |
|  | Poppy seed | 145 | 7.61 | 6.04 | 8.92 | 2.88 | [20,29] |
|  | Pumpkin seed | 185 | 14.9 | 14.3 | 15.4 | 1.11 | [30,31] |
|  | Sesame | 188 | 39.0 | 34.9 | 43.6 | 8.76 | [30,31] |
|  | Sunflower seed | 128 | 17.6 | 10.5 | 18.9 | 8.44 | [23,32] |
| Nuts | Almond | 99 | 6.61 | 4.43 | 18.8 | 14.4 | [31,32] |
|  | Almond milled | 45 | 2.53 |  |  |  | [33] |
|  | Brazil nut | 71 | 277 |  |  |  | [23] |
|  | Cashew nut | 57 | 29.0 |  |  |  | [22] |
|  | Chestnut | 22 | 1.45 |  |  |  | [22] |
|  | Hazelnut milled | 41 | 13.6 | 1.31 | 17.6 | 16.3 | [17,33] |
|  | Hazelnut | 15 | 40.6 |  |  |  | [32] |
|  | Macadamia nut roasted salted | 59 | 16.6 |  |  |  | [22] |
|  | Peanut roasted | 241 | 36.8 | 14.7 | 47.4 | 32.7 | [30-33] |
|  | Pistachio roasted | 86 | 15.7 | 8.55 | 17.0 | 8.49 | [18] |
|  | Pistachio | 65 | 24.0 | 11.1 | 31.0 | 19.9 | [18,22] |
|  | Walnut | 84 | 8.70 | 3.50 | 16.4 | 12.9 | [20,33] |
| Fruits | Apple | 121 | 1.85 | 0.15 | 2.00 | 1.85 | [18,21] |
|  | Apricot dried | 63 | 2.06 |  |  |  | [22] |
|  | Apricot | 128 | 0.55 |  |  |  | [28] |
|  | Avocado | 101 | 0.78 |  |  |  | [18] |
|  | Banana | 358 | 1.37 | 0.97 | 2.00 | 1.03 | [25,28,34] |
|  | Blackberry | 97 | 1.65 |  |  |  | [31] |
|  | Cherry | 20 | 0.17 | 0.15 | 0.19 | 0.04 | [17,31] |
|  | Citrone | 98 | 0.76 |  |  |  | [30] |
|  | Coconut | 12 | 3.23 |  |  |  | [32] |
|  | Cranberry | 43 | 0.80 |  |  |  | [28] |
|  | Currant | 66 | 0.31 |  |  |  | [22] |
|  | Date dried | 103 | 6.08 |  |  |  | [24] |
|  | Gooseberry | 55 | 1.61 |  |  |  | [32] |
|  | Grape | 294 | 0.63 | 0.15 | 0.79 | 0.64 | [24-26,28] |
|  | Grapefruit | 105 | 0.72 | 0.22 | 0.80 | 0.58 | [20,21] |
|  | Khaki | 66 | 0.98 |  |  |  | [22] |
|  | Kiwi | 207 | 1.07 |  |  |  | [21] |
|  | Mandarin | 228 | 1.03 | 0.81 | 1.24 | 0.43 | [29,30,34] |
|  | Mango | 65 | 0.25 |  |  |  | [22] |
|  | Nectarine | 254 | 1.27 | 0.62 | 2.69 | 2.07 | [18,29,34] |
|  | Orange | 209 | 1.03 | 0.06 | 2.14 | 2.08 | [29,34] |
|  | Peach | 235 | 0.84 | 0.17 | 1.04 | 0.87 | [18,20,29,34] |
|  | Pear | 302 | 1.21 | 0.65 | 2.08 | 1.43 | [29,32,34] |
|  | Pineapple | 304 | 0.88 | 0.18 | 0.91 | 0.73 | [19,20,33] |
|  | Plum | 196 | 0.83 |  |  |  | [21] |
|  | Raspberry | 169 | 0.76 | 0.20 | 0.80 | 0.60 | [20,21] |
|  | Redcurrant | 95 | 1.36 |  |  |  | [32] |
|  | Rhubarb | 199 | 0.87 |  |  |  | [21] |
|  | Strawberry | 230 | 1.01 | 0.19 | 1.25 | 1.06 | [19,21,33] |
|  | Water melone | 106 | 0.76 |  |  |  | [24] |
| Vegetables | Artichoke | 54 | 1.26 |  |  |  | [29] |
|  | Asparagus | 207 | 1.47 |  |  |  | [21] |
|  | Beetroot | 136 | 1.13 |  |  |  | [21] |
|  | Broccoli | 193 | 1.42 | 1.19 | 1.54 | 0.35 | [28,29] |
|  | Brown alga dried | 25 | 32.9 | 3.20 | 81.0 | 77.8 | [28] |
|  | Brussels sprout | 88 | 7.04 |  |  |  | [20] |
|  | Carrot juice | 197 | 1.06 |  |  |  | [34] |
|  | Carrot | 483 | 2.25 | 0.22 | 6.92 | 6.70 | [17,29-32,34] |
|  | Cauliflower | 199 | 1.32 | 1.13 | 1.50 | 0.37 | [27,30] |
|  | Celeriac | 174 | 1.39 |  |  |  | [25] |
|  | Corn | 85 | 0.79 |  |  |  | [24] |
|  | Cucumber | 359 | 0.90 | <LOD | 1.00 | 1.00 | [17,27,30-32] |
|  | Eggplant | 216 | 0.52 | 0.15 | 0.69 | 0.54 | [25,27,28] |
|  | Endive | 86 | 1.88 | 0.15 | 2.11 | 1.96 | [17,31] |
|  | Garlic | 51 | 2.63 |  |  |  | [17] |
|  | Iceberg lettuce | 61 | 2.01 |  |  |  | [33] |
|  | Kale | 110 | 4.83 |  |  |  | [24] |
|  | Kohlrabi | 191 | 1.07 |  |  |  | [21] |
|  | Lambs lettuce | 171 | 1.22 | 0.42 | 1.35 | 0.93 | [17,30,31,33] |
|  | Leek | 258 | 1.35 | 0.20 | 1.89 | 1.69 | [19-21,33] |
|  | Lettuce | 260 | 2.70 | 0.15 | 6.22 | 6.07 | [18,20,21,26,33] |
|  | Onion | 184 | 1.52 | 0.53 | 1.61 | 1.08 | [20,32] |
|  | Radish | 60 | 1.17 |  |  |  | [22] |
|  | Red alga dried | 81 | 33.7 | 10.2 | 34.3 | 24.1 | [28] |
|  | Red cabbage | 97 | 1.82 |  |  |  | [33] |
|  | Red radish | 170 | 0.69 | 0.15 | 0.76 | 0.61 | [22,24,28] |
|  | Romana lettuce | 63 | 0.65 |  |  |  | [22] |
|  | Rucola | 363 | 1.56 | 0.26 | 1.87 | 1.61 | [23-25,28,33] |
|  | Savoy cabbage | 152 | 1.49 | 0.84 | 1.80 | 0.96 | [18,22] |
|  | Spinach frozen | 153 | 1.43 | 0.73 | 1.88 | 1.15 | [18,34] |
|  | Spinach | 500 | 2.47 | 0.22 | 5.19 | 4.97 | [17,18,29,31,32,34] |
|  | Spring onion | 242 | 1.02 | 0.71 | 1.23 | 0.52 | [30,31] |
|  | Tomato | 200 | 0.74 | 0.15 | 0.82 | 0.67 | [19,21,33] |
|  | White cabbage | 244 | 1.60 | 0.42 | 2.69 | 2.27 | [19,20,22,27] |
|  | Zucchini | 213 | 0.91 | 0.21 | 0.97 | 0.76 | [20,21] |
| Legumes and  legume products | Bean green | 445 | 1.19 | 0.16 | 2.12 | 1.96 | [17,29-32,34] |
|  | Bean white dried | 88 | 8.12 |  |  |  | [20] |
|  | Chickpea flour | 4 | 6.20 |  |  |  | [91] |
|  | Lense dried | 183 | 50.1 | 34.3 | 63.6 | 29.3 | [17] |
|  | Lupine flour | 5 | 4.70 |  |  |  | [91] |
|  | Pea flour | 1 | 1.60 |  |  |  | [91] |
|  | Pea green frozen | 216 | 2.21 | 1.06 | 2.44 | 1.38 | [25-27] |
|  | Pea green | 253 | 13.2 | 0.98 | 30.2 | 29.2 | [23,28] |
|  | Soy bean | 47 | 6.22 |  |  |  | [31] |
|  | Soy flour | 5 | 19.1 |  |  |  | [91] |
|  | Tofu | 253 | 3.30 | 2.55 | 3.84 | 1.29 | [20,28,34] |
| Mushrooms | Mushroom canned | 75 | 5.45 |  |  |  | [29] |
|  | Mushroom | 196 | 11.7 | 9.08 | 13.0 | 3.92 | [22,24] |
|  | Oyster mushroom | 62 | 1.04 |  |  |  | [22] |
|  | Shiitake mushroom dried | 74 | 14.5 |  |  |  | [29] |
| Potato and  potato products | Mashed potato powder | 69 | 2.71 |  |  |  | [29] |
|  | Potato peeled | 238 | 1.00 | 0.90 | 1.10 | 0.20 | [17,32] |
| Herbs | Basil | 76 | 1.64 |  |  |  | [20] |
|  | Chive | 100 | 1.35 |  |  |  | [23] |
|  | Dill | 55 | 3.28 |  |  |  | [23] |
|  | Oregano | 45 | 1.31 |  |  |  | [23] |
|  | Parsley | 228 | 1.54 | 1.50 | 1.58 | 0.08 | [24,28] |
|  | Rosemary | 64 | 2.82 |  |  |  | [23] |
| Spices and seasonings | Mustard | 52 | 22.5 |  |  |  | [17] |
|  | Pepper | 214 | 10.1 | 8.52 | 11.9 | 3.39 | [31,34] |
|  | Sojasauce | 101 | 2.51 |  |  |  | [18] |
| Fat and oil | Corn oil | 90 | 2.12 |  |  |  | [18] |
|  | Margarine | 120 | 2.06 |  |  |  | [23] |
|  | Olive oil | 96 | 2.29 |  |  |  | [24] |
|  | Pumpkin seed oil | 69 | 0.99 |  |  |  | [28] |
|  | Rapeseed oil | 95 | 2.04 |  |  |  | [23] |
|  | Sunflower oil | 69 | 1.85 |  |  |  | [18] |
| Milk and dairy products | Cream cheese | 57 | 2.76 | <LOQ | 2.81 | 2.81 | [18] |
|  | Curd | 36 | 4.73 | 3.50 | 4.80 | 1.30 | [18] |
|  | Gouda | 176 | 15.3 | 14.6 | 16.1 | 1.44 | [17,18] |
|  | Sheep cheese | 134 | 8.34 |  |  |  | [23] |
|  | Milk | 122 | 1.44 |  |  |  | [18] |
| Egg | Egg | 153 | 22.1 | 21.1 | 24.2 | 3.12 | [24,26] |
| Fish and seafood | Cod liver canned | 30 | 57.5 |  |  |  | [26] |
|  | Cod | 80 | 24.1 |  |  |  | [34] |
|  | Crab | 134 | 45.4 | 41.3 | 48.4 | 7.13 | [18,24,32] |
|  | Eel smoked | 32 | 28.6 |  |  |  | [26] |
|  | Eel | 90 | 22.3 | 19.8 | 22.6 | 2.84 | [17,21] |
|  | Herring | 260 | 30.7 | 27.4 | 34.5 | 7.12 | [24,30,33] |
|  | Mussels | 49 | 58.9 |  |  |  | [20] |
|  | Pangasius | 220 | 10.3 | 10.1 | 10.6 | 0.52 | [30,31] |
|  | Plaice frozen | 121 | 31.5 |  |  |  | [20] |
|  | Plaice | 5 | 0.00 |  |  |  | [92] |
|  | Pollack | 120 | 20.9 |  |  |  | [23] |
|  | Rainbow trout | 220 | 15.6 | 12.2 | 18.8 | 6.67 | [23,32] |
|  | Redfish | 279 | 45.2 | 37.9 | 48.1 | 10.2 | [17,18] |
|  | Salmon | 227 | 15.7 | 13.5 | 18.6 | 5.10 | [17,29] |
|  | Shrimp | 174 | 23.8 | 23.3 | 24.4 | 1.16 | [20,28] |
|  | Sword fish | 55 | 55.2 |  |  |  | [26] |
|  | Trout smoked | 63 | 19.7 |  |  |  | [32] |
|  | Tuna canned | 62 | 72.6 |  |  |  | [24] |
|  | Tuna | 226 | 71.7 | 63.9 | 81.8 | 17.9 | [26,28,31] |
| Meat and meat products | Beef | 629 | 8.00 | 6.51 | 8.69 | 2.18 | [17,22,28,34] |
|  | Boar | 72 | 18.8 |  |  |  | [28] |
|  | Deer | 178 | 6.55 | 5.26 | 7.61 | 2.35 | [19,21] |
|  | Duck | 373 | 21.6 | 19.4 | 25.7 | 6.32 | [17,27] |
|  | Goat | 56 | 7.08 |  |  |  | [20] |
|  | Goose | 247 | 13.9 |  |  |  | [27] |
|  | Lyoner sausage | 97 | 7.28 |  |  |  | [32] |
|  | Pork | 207 | 12.6 | 11.3 | 13.6 | 2.39 | [19,21] |
|  | Rabbit | 405 | 11.0 | 10.1 | 13.2 | 3.12 | [19,27,31] |
|  | Salami | 151 | 17.5 |  |  |  | [29] |
|  | Turkey | 314 | 10.4 | 10.0 | 11.3 | 1.25 | [28,32] |
|  | Veal | 304 | 9.50 | 7.66 | 10.3 | 2.63 | [18,24] |
|  | Veal chop | 1 | 9.20 |  |  |  | [18] |
|  | Veal escalope | 1 | 9.00 |  |  |  | [18] |
|  | Veal knuckle | 1 | 9.00 |  |  |  | [18] |
|  | Veal nut | 1 | 11.0 |  |  |  | [18] |
|  | Veal shoulder | 1 | 10.0 |  |  |  | [18] |
| Offal | Beef kidney | 337 | 123 | 118 | 154 | 36.4 | [26,34] |
|  | Beef liver | 284 | 35.2 | 33.6 | 38.3 | 4.76 | [17,26] |
|  | Chicken liver | 107 | 52.8 |  |  |  | [30] |
|  | Lamb liver | 94 | 43.2 |  |  |  | [19] |
|  | Pork kidney | 156 | 221 | 220 | 223 | 3.54 | [18,26] |
|  | Pork liver | 164 | 55.9 | 51.3 | 64.1 | 12.8 | [19,20,26] |
|  | Veal kidney | 45 | 98.6 |  |  |  | [26] |
|  | Veal liver | 204 | 41.8 | 39.4 | 43.2 | 3.77 | [19,24,26] |
| Sweets | Cacao powder | 116 | 25.6 | 8.00 | 31.4 | 23.4 | [24,32] |
|  | Chocolate | 13 | 32.6 |  |  |  | [32] |
|  | Dark chocolate | 259 | 10.9 | 8.06 | 13.8 | 5.76 | [24,26] |
|  | Honey | 133 | 0.17 |  |  |  | [22] |
|  | Lakritze | 100 | 3.72 |  |  |  | [32] |
|  | Marzipan | 48 | 1.99 |  |  |  | [29] |
| Beverages | Apple juice | 329 | 0.71 | 0.23 | 0.98 | 0.75 | [19,20,29,32] |
|  | Beer | 318 | 1.34 | 1.17 | 2.00 | 0.83 | [22,34] |
|  | Beetroot juice | 45 | 1.00 |  |  |  | [21] |
|  | Black tea | 174 | 0.30 | 0.20 | 0.38 | 0.18 | [26,34] |
|  | Coffee | 59 | 0.50 |  |  |  | [19] |
|  | Dark beer | 67 | 0.01 |  |  |  | [22] |
|  | Grape juice | 379 | 0.57 | 0.50 | 0.67 | 0.17 | [23,34] |
|  | Grapefruit juice | 65 | 1.15 |  |  |  | [29] |
|  | Green tea | 189 | 0.24 | 0.15 | 0.32 | 0.17 | [26,34] |
|  | Herbs tea | 74 | 0.26 | 0.19 | 0.37 | 0.18 | [24] |
|  | Orange juice | 313 | 0.83 | 0.49 | 1.30 | 0.81 | [25,28,33] |
|  | Pear juice | 116 | 0.76 |  |  |  | [20] |
|  | Peppermint tea | 71 | 0.46 |  |  |  | [32] |
|  | Pineapple juice | 51 | 1.02 |  |  |  | [29] |
|  | Redbush tea | 61 | 4.33 |  |  |  | [32] |
|  | Tomato juice | 90 | 0.69 |  |  |  | [26] |

* blanks mean no data available
